# Co-designing Entrustable Professional Activities in General Practitioner’s training: a participatory research study

**DOI:** 10.1186/s12909-024-05530-y

**Published:** 2024-05-17

**Authors:** Vasiliki Andreou, Sanne Peters, Jan Eggermont, Birgitte Schoenmakers

**Affiliations:** 1 Academic Centre for General Practice, Department of Public Health and Primary Care, KU Leuven, Leuven, Belgium; 2 School of Health Sciences, Faculty of Medicine, Dentistry and Health Sciences, The University of Melbourne, Melbourne, Australia; 3 Department of Cellular and Molecular Medicine, KU Leuven, Leuven, Belgium; 4 Department of Public Health and Primary Care, KU Leuven, Box 7001, Kapucijnenvoer 7, Leuven, 3000 Belgium

**Keywords:** Postgraduate medical education, Curriculum design, EPA assessment, GP Training, Workplace-based assessment

## Abstract

**Background:**

In medical education, Entrustable Professional Activities (EPAs) have been gaining momentum for the last decade. Such novel educational interventions necessitate accommodating competing needs, those of curriculum designers, and those of users in practice, in order to be successfully implemented.

**Methods:**

We employed a participatory research design, engaging diverse stakeholders in designing an EPA framework. This iterative approach allowed for continuous refinement, shaping a comprehensive blueprint comprising 60 EPAs. Our approach involved two iterative cycles. In the first cycle, we utilized a modified-Delphi methodology with clinical competence committee (CCC) members, asking them whether each EPA should be included. In the second cycle, we used semi-structured interviews with General Practitioner (GP) trainers and trainees to explore their perceptions about the framework and refine it accordingly.

**Results:**

During the first cycle, 14 CCC members agreed that all the 60 EPAs should be included in the framework. Regarding the formulation of each EPAs, 20 comments were given and 16 adaptations were made to enhance clarity. In the second cycle, the semi-structured interviews with trainers and trainees echoed the same findings, emphasizing the need of the EPA framework for improving workplace-based assessment, and its relevance to real-world clinical scenarios. However, trainees and trainers expressed concerns regarding implementation challenges, such as the large number of EPAs to be assessed, and perception of EPAs as potentially high-stakes.

**Conclusion:**

Accommodating competing stakeholders’ needs during the design process can significantly enhance the EPA implementation. Recognizing users as experts in their own experiences empowers them, enabling a priori identification of implementation barriers and potential pitfalls. By embracing a collaborative approach, wherein diverse stakeholders contribute their unique viewpoints, we can only create effective educational interventions to complex assessment challenges.

## Introduction

In recent years, the landscape of medical education has significantly transformed due to increasing demands of public accountability and changing patient needs. In response to these evolving demands, competency-based medical education (CBME) has emerged. CBME has been gaining popularity in medical education programs [1]. In a CBME paradigm, medical curricula are structured based on predefined competencies that physicians should have acquired upon completion of the program [2, 3]. Despite the theoretical underpinnings of CBME, its implementation has encountered various obstacles [4]. Particularly, assessing competencies in real clinical environments has been a major barrier in the effective integration of CBME into medical education systems [5]. Recognizing this challenge, the concept of Entrustable Professional Activities (EPAs) has emerged.

EPAs are essentially tasks or activities that medical professionals should be able to perform competently and independently by the time they complete their training [6, 7]. EPAs are used to assess a learner’s ability to integrate and apply the necessary competencies in real-world clinical practice. They necessitate evaluating a learner’s progress and readiness for independent practice by observing their performance in these key professional activities in clinical practice [8]. The term “entrustable” indicates that, upon graduation or completion of a specific training period, a supervising physician or mentor should be able to entrust a medical graduate with these activities without direct supervision, considering them proficient and safe for the patients to perform these tasks independently [9, 10].

Considering the immense potential, integration and implementation of EPAs has gained rapid momentum, across various health professions and medical specialties [11, 12]. Despite this progress, a significant gap notably persists, when it comes to accommodating competing needs of curriculum designers and those of users in practice, namely trainers and trainees [13]. While the promise of EPAs in facilitating CBME is promising, there is lack of comprehensive evidence incorporating users’ perceptions during the design phase [8, 11, 14]. Therefore, the aim of this study was to design an EPA framework for workplace-based assessment by actively involving clinical educators, trainees and trainers throughout the process.

## Methods

### Setting and participants

This study took place in the interuniversity postgraduate General Practitioner’s (GP) Training, Belgium. To standardize GP Training across Flanders, four Flemish universities (KU Leuven, Ghent University, University of Antwerp, and the Flemish Free University of Brussels) collaboratively developed a postgraduate training program. This training program consists of three different training-phases and rotations, spread through three years, two rotations are in a GP practice, while one takes place at a hospital setting.

The GP Training is overseen by the Interuniversity Centre for GP Training (ICGPT). The ICGPT plays a pivotal role in coordinating and managing various aspects of the curriculum. Among its key responsibilities, the ICGPT oversees the allocation of clinical internships, conducts examinations, facilitates regular meetings between trainees and trainers, and maintains trainees’ learning electronic (e-) portfolios.

In 2018, the ICGPT initiated a shift towards CBME. The rationale of CBME was introduced in the curriculum by integrating first the CanMEDS roles. To facilitate this transition, two clinical competence committees (CCCs), comprising medical doctors and clinical educators from the four universities were appointed. These CCCs were tasked with coordinating workplace-based learning, and curriculum and assessment, respectively.

To align the curriculum with the patient needs in primary care, the two CCCs designated and defined ten different care contexts characteristic of primary care (i.e. short-term care, chronic care, emergency care, palliative care, elderly care, care for children, mental healthcare, prevention, gender related care, and practice management). Subsequently, in 2022, we initiated the process of designing specific EPAs for the GP Training. The EPAs aimed to facilitate and improve workplace-based assessment. These two CCCs participated in the design process, while trainers and trainees were invited to share their opinion as well.

### Designing the EPA framework

**Fig. 1:**
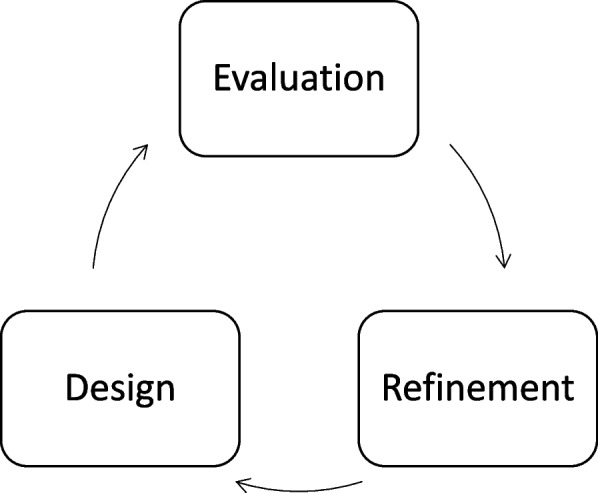
Three design phases for designing the EPA framework

**Fig. 2:**
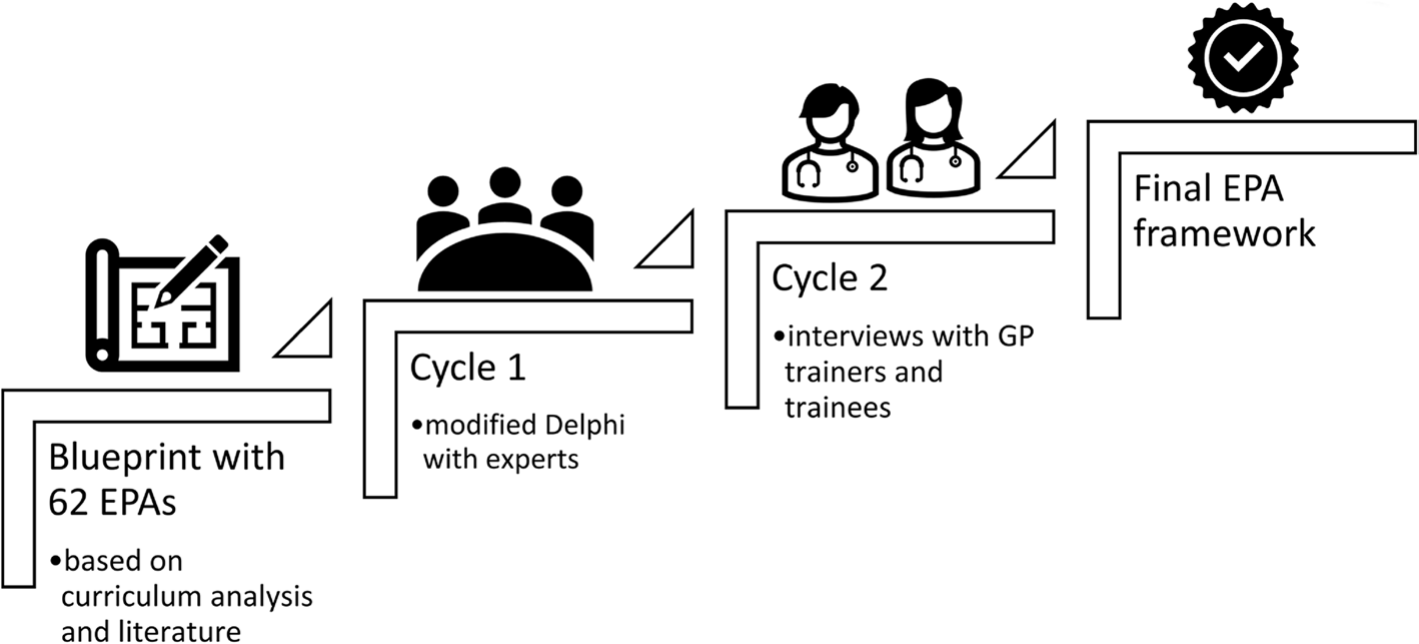
Process for developing the EPA framework based on participatory design research

The design of the EPA framework was based on participatory research design to engage different stakeholders [15]. Participatory research design is a community-based methodology aiming to create solutions for and with the people who are involved [15]. This iterative research approach encompassed three fundamental design-stages in a circular relationship, namely design, evaluation and refinement (Fig. 1). We executed two distinct iterative cycles, each with a specific group of stakeholders (Fig. 2). In cycle 1, we focused on CCCs, fostering discussions and validating the framework. In cycle 2, we involved clinical trainers and trainees, ensuring cross-validation. In the following section, we describe each iterative cycle, indicated as cycle 1 and as cycle 2, respectively.

In cycle 1, after reviewing relevant literature, we developed a blueprint of 60 EPAs corresponding to the ten different care contexts, already integrated in the curriculum [9, 10]. By doing so, we wanted to ensure practical applicability and relevance of our framework within the established educational environment. Afterwards, we linked all EPAs to the CanMEDS competency framework [16]. We defined competencies as broad statements that describe knowledge, skills and attitudes that GP trainees should achieve during the different training phases [17]. The CanMEDS framework identifies and describes different competencies for patient-centred care, and comprises seven different roles: medical expert, communicator, collaborator, leader, health advocate, scholar, and professional. By linking EPAs to CanMEDS, we constructed a matrix that served as a structured guide for integrating the EPAs in the workplace. Also, together with the CCCs we defined behavioural and cognitive criteria to anchor entrustment levels [9]. These criteria described required knowledge, skills, and attitudes in order for an EPA to be entrusted.

**Fig. 3:**
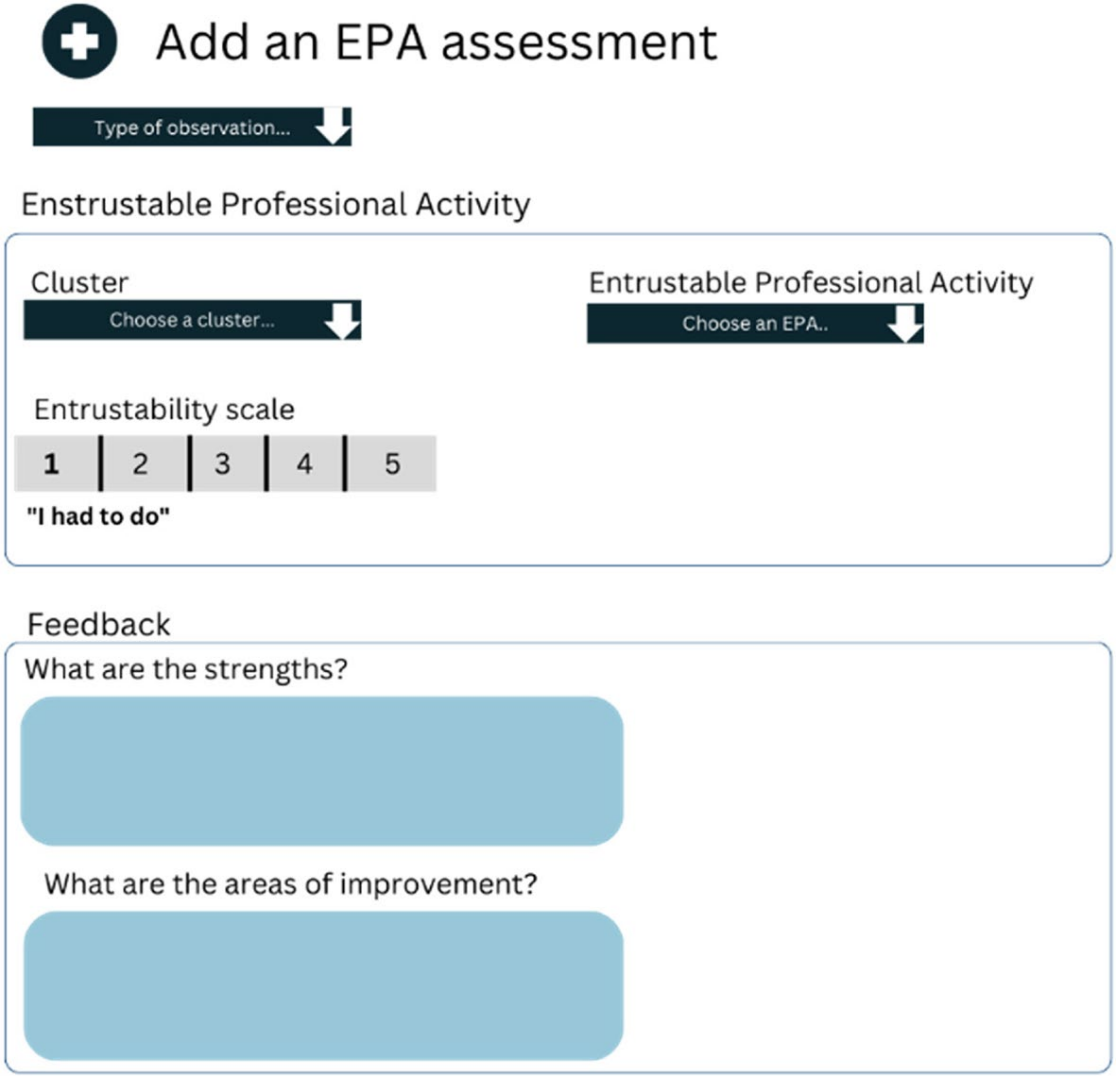
Example of Clinical Practice Feedback form available in the e-portfolio

**Fig. 4:**
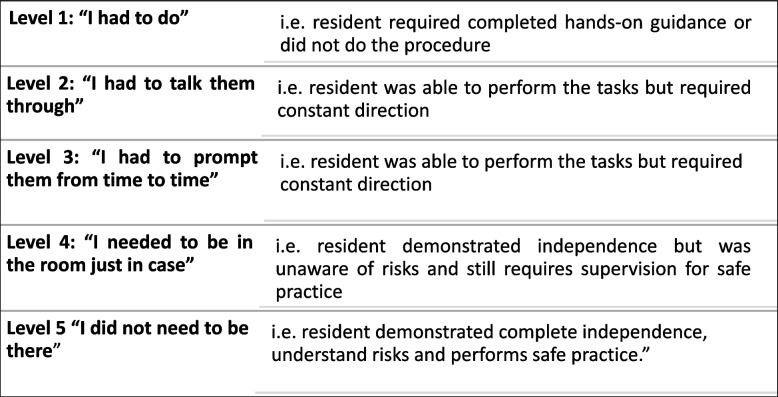
Five levels of entrustment based on the O-SCORE scale [19]

In cycle 2, we aimed at operationalising the EPAs, cross validating them by interviewing trainers and trainees, and deciding entrustment levels. Specifically, to operationalise the EPAs, we developed an assessment form, called Clinical Practice Feedback form (Fig. 3). We chose to link EPA assessments not only to direct and video observations, but also for case-based discussions. Additionally, we agreed upon entrustment levels and the entrustability scale. Entrustment was anchored on criteria that were defined along the EPAs. We decided to use the Ottawa Surgical Competency Operating Room Evaluation (O-SCORE) for validity and reliability reasons (Fig. 4) [18]. The Ottawa scale requires assessors to describe how much supervision they provided to trainees while performing a specific EPA. Concretely, the scale comprises five levels of performance ranging from trainers taking over the activity to trainees performing the activity without supervision (Fig. 3) [18].

### Data collection and analysis

In cycle 1, we evaluated the EPA blueprint by employing a modified Delphi methodology, with two rounds [19]. We invited members of the two CCCs (*N* = 14) to give feedback on the EPA blueprint via e-mail and during meetings, scheduled by the ICGPT. Members were asked whether they thought each EPA was necessary for workplace-based assessment and needed to be included in the framework. They were also encouraged to give feedback regarding the formulation of the EPAs. Once we gathered all the comments, we refined the blueprint and sent it back to the CCC members. In cycle 2, we interviewed two trainers and two trainees using semi-structured interviews and following the ‘think-aloud protocol’ [20–22], where we asked them whether each EPA was necessary and whether they were comprehensible for workplace-based assessment. Participants were required to articulate their thoughts while reading the EPA framework. This enabled us to gain insights into their thought processes and perspectives [22].

Data collection took place from February 2022 until September 2022. For quantitative data analysis we calculated descriptive statistics of consensus rates using SPSS 27 (IBM SPSS Statistics 27). We analysed qualitative data from CCCs members using content analysis on Microsoft Excel. For analysing data from the interviews with the trainers and trainees, we first verbatim transcribed the interviews, and, then, analysed the data using thematic analysis in NVivo (QSR International) [23, 24]. Qualitative data were analysed by two researchers separately to achieve triangulation, while a third researcher was consulted, when discrepancies arose [25].

### Reflexivity and research team

The research team was composed of members with different backgrounds. Two members had a background in education, while the other two members had a background in biomedical sciences and general practice. All authors had research training and experience in medical education research. Methodological and design decisions were in line with the available literature. We predefined methodological steps before commencing the study. To ensure adherence to our design stages, we maintained a detailed logbook to document systematically progression and modifications from our initial protocol. We regularly discussed the results to ensure that our interpretations were close to the data.

## Results

In cycle 1, fourteen members of the CCCs gave feedback on the list of 60 EPAs. In the first feedback round, all members agreed that all 60 EPAs were required in the framework. Twenty comments were given regarding the formulation of the EPAs and 16 adaptations were made based on the new suggestions. Comments regarding the formulation were about the use of certain words in order to make the framework understandable. In the second feedback round, consensus was reached on the formulation of the EPAs (Table 1).

**Table 1:** List of EPAs for the Flemish GP Training

Types of care	EPA		Consensus rate	
Gender-Related Care	Guide patients in making informed decisions about temporary or permanent contraception.	100%		
	Conduct expert preconception consultations, including explaining the possibilities and limitations of genetic counselling;	100%		
	Assist in normally progressing pregnancies in their various dimensions (with support);	100%		
	Guide the patient (and partner) and provide expert advice in case of unwanted pregnancy (with support);	100%		
	Provide expert guidance to the patient in the postpartum period (with support);	100%		
	Diagnose and expertly treat STDs and gynaecological infections and provide information about them, with special attention to risk groups;	100%		
	Conduct expert medical history and clinical examination for menopausal symptoms and explain and guide menopausal symptoms;	100%		
	Expertly interpret results of a cervico-vaginal smear;	100%		
	Conduct a sexological medical history and address sexual problems;	100%		
	Diagnose and expertly treat LUTS (Lower Urinary Tract Symptoms) and sexual problems.	100%		
	Expertly inform the patient regarding targeted prostate cancer screening.	100%		
	Perform expert skills: gynaecological examination, pregnancy examination, insertion of an IUD (intra-uterine device), rectal examination and interpretation.	100%		
Short episode care	Clarify the help request (Ideas, Concerns, Expectations), take a focused medical history (aimed at diagnostic landscape), conduct a focused physical examination, reach a diagnosis or ‘working hypothesis’.		100%	
	Decide if additional diagnostics are necessary (what, where), request/perform them motivated (evidence-based and cost-conscious) or proceed directly to ‘treatment and management’.		100%	
	Establish a treatment and management plan (shared decision-making), inform about it (in understandable language), and advise (prognosis and therapy, self-care).		100%	
	Record data in the electronic medical record and code (ICPC, maintain problem list) to promote continuity of care, make information accessible for research, statistics, and evaluate own care.		100%	
	Complete an episode (evaluate treatment/management, adjust, discuss additional tests, evaluate non-pharmacological advice, discuss prognosis and expectations) and close.		100%	
	Assess the help request and determine the type of contact (phone consultation, in-person consultation, home visit) (in consultation with the patient and reception, based on knowledge of epidemiology and disease patterns).		100%	
Emergency care	Determine the patient’s condition with acute illness (ABCDE) and perform necessary interventions.		100%	
	Handle prioritized consultations and visits according to urgency.		100%	
	Document findings, diagnosis, and management clearly and communicate them effectively.		100%	
Chronic care	Treat, monitor, and organize care for patients with chronic conditions (diabetes, cardiovascular diseases, oncological issues, asthma/COPD).		100%	
	Record and share data with co-practitioners and adjust the management.		100%	
	Identify the moment when a chronic illness requiring treatment and monitoring occurs.		100%	
Elderly patient care	Determine if complex issues are present and consider this in the management.		100%	
	Discuss early choices regarding additional diagnostics and/or treatment considering the personal wishes and life expectancy of the elderly individual.		100%	
	Improve and monitor the safety of elderly care in practice, including periodic medication reviews and fall prevention programs.		100%	
Care for Children	Conduct consultations with acutely ill children (0–12 years).		100%	
	Prescribe medication for children, determining appropriate dosage and administration.		100%	
	Support newborns and their parents (with assistance).		100%	
	Determine appropriately when a consultation should be conducted with the child alone and when, or in what manner, parents should be involved.		100%	
	Handle the rights of the child and parents adequately, even in specific situations (divorce, joint custody, blended families, etc.).		100%	
Mental Health	Guide patients with anxiety and mood disorders.		100%	
	Initiate necessary treatment for patients with anxiety and mood disorders.		100%	
	Assess the danger of suicide for the patient and their environment and provide necessary help.		100%	
	Identify personality traits and disorders, define their implications for healthcare, and advise the patient to seek appropriate guidance if needed.		100%	
	Establish a working hypothesis for substance abuse issues, explain and treat step by step.		100%	
	Utilize the Electronic Medical Record to discover patterns in complaints and influencing factors.		100%	
	Conduct a referral conversation with a patient with substance abuse issues.		100%	
	Discuss the impact of complaints on employment participation in (potential) long-term disability cases.		100%	
	Provide long-term support and maintain control in patients with severe substance abuse issues.		100%	
Palliative care	Practice early care planning and advance directives.		100%	
	Initiate end-of-life discussions at the appropriate time.		100%	
	Address specific problems in the palliative/terminal phase.		100%	
	Provide or contribute to personal continuity of care to maintain practice continuity.		100%	
Prevention	Recognize intervention possibilities for prevention.		100%	
	Explore lifestyle choices of patients when indicated and discuss possibilities for change.		100%	
	Provide advice on various preventive activities.		100%	
	Identify individuals and patients with increased health risks.		100%	
	Contribute to programmatic prevention efforts.		100%	
	Participate in population screening programs as agreed upon by the professional group and the government.		100%	
	Investigate hereditary diseases in a patient’s family and discuss whether screening or diagnostics are necessary, and refer if appropriate.		100%	
	Identify other healthcare providers who can play a role in prevention and collaborate with them.		100%	
Practice Management	Have a personal and practice vision for general practitioner care and develop collaboration in the practice.		100%	
	Develop and implement an improvement plan.		100%	
	Observe, supervise, and communicate with practice staff when performing reserved acts.		100%	
	Contribute to patient safety by reporting, analysing, and addressing patient safety issues.		100%	
	Understand the financing and business management of the general practice, handle social security regulations and codes for payment and other systems for billing or reimbursement, and advise patients on the financial consequences of medical treatment/diagnostics.		100%	
	Represent the practice in multidisciplinary meetings and discussions with external parties.		100%	
	Align practice management and information provision with the needs of the patient population.		100%	

In cycle 2, we interviewed two trainers and two trainees. CCC members, trainers, and trainees agreed that all EPAs should be included in the framework. From these interviews, we identified three themes. Table 2 presents these three themes alongside their subthemes. Necessity of EPAs was the first theme and included shared mindsets about necessity of EPAs in order to improve workplace-based assessment and difficulties with interpreting the CanMEDS roles.

**Table 2:** Themes from the interviews with trainers and trainees

Theme	Subtheme
Necessity of EPAs	• Improving workplace-based assessment • Difficulties in workplace-based assessment based on the CanMEDS roles
Relevance of EPAs to clinical practice	• Intuitive formulation of EPAs • Understandable language
Challenges in implementation	• Large number of EPAs • Use of e-portfolio for high-stakes assessments • Limited functionalities of current e-portfolio

“*The EPAs are better than the CanMEDS. My trainer and I often do not know what we have to assess…He (the trainer) sometimes gives the same feedback for multiple roles*.” (trainee 1).

Second theme was about the relevance of EPAs to clinical practice. Users thought that the EPA framework could easily be linked to their clinical work, promoting assessment and feedback opportunities. They agreed that EPAs were understandable and formulated in intuitive language for clinical work.

“*I think that it (the EPA framework) is quite intuitive. I can see a lot of links between the EPAs and my daily practice*.” (trainer 2).

*I like the (EPA) framework. My trainer and I already discuss some of these (activities) during our weekly feedback session*. (trainee 2)

Third theme included challenges in implementation of EPAs, regarding the large number of EPAs, perception of high-stakes assessment within an e-portfolio, and limitations inherent to the current e-portfolio. First, users expressed their concern regarding the large number of EPAs. They indicated that only a limited number might be feasible because of time constraints in the clinical workplace. Also, users thought that due to the large number of EPAs, trainees would “pick and choose” EPAs where they had performed well. Along with limited functionalities of the current e-portfolio, they indicated that EPAs might be used as showcasing performance instead for workplace-based assessment and feedback purposes. Mainly trainees expressed hesitation to document EPAs where they would need further improvement. They perceived the e-portfolio as a tool more suitable for high-stakes assessments rather than for feedback purposes.

“*The list (of EPAs) is quite extensive… I do want to have a nice portfolio, so for sure I will try to include as many as possible. In case something happens (in my curriculum), I want to show how well I have been performing*.” (trainee 1).

“*I normally do not include patient cases that went wrong in my portfolio. Because different people have access to it (the e-portfolio).”* (trainee 2).

## Discussion

The aim of this study was to design an EPA framework by actively engaging and collaborating with different stakeholders. To be established as a “good” assessment framework, EPAs should be acceptable by the different stakeholders involved in the assessment process, such as curriculum designers, trainees and trainers [26, 27]. Incorporating their opinions and understanding their different needs must be integral to the design process. However, literature regarding EPAs design has mainly focused on experts’ opinion, neglecting users in practice [8].

From our findings, it becomes apparent that direct involvement and communication among diverse stakeholders are crucial for designing a useful for everyone EPA assessment framework. When various groups are involved in developing educational interventions, competing needs can be optimally addressed [28]. This optimization fosters a cohesive approach, ensuring high applicability rates and effectiveness, when the EPA framework is used in practice. The need for users’ involvement in the development process is currently demonstrated in the most recent EPA literature [29, 30]. Users’ involvement promotes common language and expectations, enhancing the clarity and effectiveness of EPA interventions, and, most importantly, empowers the users themselves by acknowledging their perspectives [31]. Ultimately, trainees and trainers are the ones using the EPA assessment frameworks during daily clinical practice, and are potentially confronted with unforeseen obstacles.

Additionally, users’ involvement in the process can help to identify potential implementation challenges [32, 33]. Our findings indicate differences in opinions regarding implementation of EPAs. In contrast to the CCC members, users expressed their concerns about the large number of EPAs included in the framework. They were particularly concerned about how to use sufficiently and adequately EPA assessments, while juggling clinical work. This concern echoes findings from other studies as well, related to the assessment burden [34]. In particular, when challenges in assessment processes arise in the clinical workplace, assessment is most probably not performed as intended [35].

Furthermore, our results illustrate tensions between assessment of learning and assessment for learning. Although the EPA assessments aim to better prepare trainees for clinical practice, users suggested that the purpose of the EPAs might not be explicit for everyone. Since EPAs are a form of assessment, they could potentially lead to strategic behaviours of documenting successful EPAs, and, therefore, creating a fragmented idea about trainees’ performance in clinical practice. Additionally, the use of the current e-portfolio for high-stakes assessments only adds to this tension. Especially, trainees were not comfortable with sharing performance evidence for improvement, because they perceived the stakes as high [36]. The dilemma between learning versus performing has been the Achilles point in workplace-based assessment [37]. The lines between assessment and feedback seem to be also blurred in EPAs [38, 39].

Involving users during the design process can lead not only to early adaptations and refinement of EPAs, but also to better allocation of resources. In order to ensure successful implementation of EPAs, it is essential to recognize the central role of both trainers and trainees. Future research should focus on training programs designed to equip faculty, trainers, and trainees with a profound understanding of EPAs. Users in practice need rigorous training covering EPA principles, assessment techniques, and feedback strategies [40]. Moreover, fostering a culture of interdisciplinary collaboration among stakeholder groups is imperative. Encouraging review of assessment tools and facilitating the exchange of opinions during designprocesses can significantly enhance the overall quality of EPA frameworks, and, even more broadly, of workplace-based assessment practices.

Although EPAs are a valuable framework for assessing competencies in workplace settings, integrating other assessment tools is crucial to capture the full spectrum of skills needed to meet patient needs. Future research should focus on combining EPAs with other assessment methods, such as simulation-based assessments, either with standardized patients or with virtual reality, that would allow trainees to demonstrate their clinical and interpersonal skills within safe, controlled environments that closely replicate challenging patient scenarios [41]. Additionally, incorporating multisource feedback and continuous portfolio assessments could offer a comprehensive view of a trainee’s performance across various settings and interactions [42, 43]. Together, these methods would enhance the EPA framework, ensuring a comprehensive assessment of all essential competencies that future physicians should acquire.

## Limitations

We need to acknowledge several limitations in this study. First, in medical education research, users’ involvement prerequisites a degree of experience with a specific subject. In our study, we involved users in the early design process of the EPA framework. Although we are aware of this limitation, we intentionally and consciously chose a participatory research design. We believe that users are experts in their own experience, and that they hold the knowledge and capabilities to be involved as partners in the development process. Second, our study involved a low number of users due to difficulties in recruitment. This might have led to recruiting participants who are fully engaged in the educational practices of the GP Training. Nevertheless, our findings are rooted in two methodologies, namely a modified Delphi method and semi-structured interviews. Therefore, we used triangulation to verify our results [25]. Finally, although workshops are mostly commonly in co-design studies [44], our study coincided with the last COVID-19 lockdown, necessitating adjustments. To cope with these challenges and uncertainties, we opted for methods that were the most feasible for our participants at that moment. Despite these challenges, the contributions from all stakeholders were invaluable, particularly in exploring potential implementation and evaluation issues.

## Conclusion

For EPAs to be successful, they need to be acceptable as an assessment framework by different stakeholders’ groups. Accommodation of competing stakeholders’ needs during the design process is crucial for enhancing acceptability and effectiveness during implementation. Our findings highlight the significance of collaborative efforts to design EPAs, emphasizing its potential to empower users, identify implementation barriers, and pinpoint unintended consequences. Through this collaborative approach, wherein diverse stakeholders contribute their perspectives, we can create effective educational solutions to complex assessment challenges.

## Data Availability

The datasets used and/or analysed during the current study are available from the corresponding author on reasonable request.

## Abbreviations

GP: General Practitioner
CBME: competency-based medical education
EPA: Entrustable Professional Activity
CanMEDS: Canadian Medical Education Directives for Specialists
ICGPT: Interuniversity Centre for GP Training
CCC: clinical competence committee

## References

[1] Frank JR, Snell LS, Cate OT, Holmboe ES, Carraccio C, Swing SR (2010). Competency-based medical education: theory to practice. Med Teach.

[2] Iobst WF, Sherbino J, Cate OT, Richardson DL, Dath D, Swing SR (2010). Competency-based medical education in postgraduate medical education. Med Teach.

[3] Frank JR, Snell L, Englander R, Holmboe ES (2017). Implementing competency-based medical education: moving forward. Med Teach.

[4] Nousiainen MT, Caverzagie KJ, Ferguson PC, Frank JR (2017). Implementing competency-based medical education: what changes in curricular structure and processes are needed?. Med Teach.

[5] Lockyer J, Carraccio C, Chan M-K, Hart D, Smee S, Touchie C (2017). Core principles of assessment in competency-based medical education. Med Teach.

[6] Ten Cate O, Scheele F (2007). Competency-based postgraduate training: can we bridge the gap between theory and clinical practice?. Acad Med.

[7] Carraccio C, Englander R, Gilhooly J, Mink R, Hofkosh D, Barone MA (2017). Building a framework of entrustable professional activities, supported by competencies and milestones, to bridge the educational continuum. Acad Med.

[8] Ten Cate O, Chen HC, Hoff RG, Peters H, Bok H, van der Schaaf M (2015). Curriculum development for the workplace using entrustable professional activities (EPAs): AMEE guide no. 99. Med Teach.

[9] Ten Cate O, Taylor DR (2021). The recommended description of an entrustable professional activity: AMEE guide no. 140. Med Teach.

[10] Carraccio C, Martini A, Van Melle E, Schumacher DJ (2021). Identifying core components of EPA implementation: a path to knowing if a complex intervention is being implemented as intended. Acad Med.

[11] de Graaf J, Bolk M, Dijkstra A, van der Horst M, Hoff RG, Ten Cate O (2021). The implementation of entrustable professional activities in postgraduate medical education in the Netherlands: rationale, process, and current status. Acad Med.

[12] Keeley MG, Bray MJ, Bradley EB, Peterson CM, Waggoner-Fountain LA, Gusic ME (2022). Fidelity to best practices in EPA implementation: outcomes supporting use of the core components framework from the University of Virginia entrustable professional activity program. Acad Med.

[13] St-Onge C, Boileau E, Langevin S, Nguyen LHP, Drescher O, Bergeron L (2022). Stakeholders’ perception on the implementation of developmental progress assessment: using the theoretical domains framework to document behavioral determinants. Adv Health Sci Educ.

[14] Taylor DR, Park YS, Egan R, Chan MK, Karpinski J, Touchie C (2017). EQual, a novel rubric to evaluate entrustable professional activities for quality and structure. Acad Med.

[15] Wallerstein N, Duran B (2010). Community-based participatory research contributions to intervention research: the intersection of science and practice to improve health equity. Am J Public Health.

[16] Frank JR, Snell L, Sherbino J. CanMEDS 2015 Physician competency framework. Ottawa: Royal College of Physicians & Surgeons of Canada; 2015.

[17] Harden RM (2002). Learning outcomes and instructional objectives: is there a difference?. Med Teach.

[18] Gofton WT, Dudek NL, Wood TJ, Balaa F, Hamstra SJ (2012). The Ottawa surgical competency operating room evaluation (O-SCORE): a tool to assess surgical competence. Acad Med.

[19] de Villiers MR, de Villiers PJ, Kent AP (2005). The Delphi technique in health sciences education research. Med Teach.

[20] Patton MQ, Fund RECM. Qualitative research & evaluation methods. SAGE Publications; 2002.

[21] Sargeant J (2012). Qualitative research part II: participants, analysis, and quality assurance. J Graduate Med Educ.

[22] Ericsson KA, Simon HA (1998). How to study thinking in everyday life: contrasting think-aloud protocols with descriptions and explanations of thinking. Mind Cult Act.

[23] Lumivero. NVivo (Version 14). 2023. www.lumivero.com.

[24] Krippendorff K. Content analysis: an introduction to its methodology. Sage; 2018.

[25] Carter N, Bryant-Lukosius D, DiCenso A, Blythe J, Neville AJ (2014). The use of triangulation in qualitative research. Oncol Nurs Forum.

[26] Norcini J, Anderson B, Bollela V, Burch V, Costa MJ, Duvivier R (2011). Criteria for good assessment: consensus statement and recommendations from the Ottawa 2010 conference. Med Teach.

[27] Norcini J, Anderson MB, Bollela V, Burch V, Costa MJ, Duvivier R (2018). 2018 Consensus framework for good assessment. Med Teach.

[28] Göttgens I, Oertelt-Prigione S (2021). The application of human-centered design approaches in health research and innovation: a narrative review of current practices. JMIR Mhealth Uhealth.

[29] Bonnie LHA, Visser MRM, Bont J, Kramer AWM, van Dijk N (2019). Trainers’ and trainees’ expectations of entrustable professional activities (EPAs) in a primary care training programme. Educ Prim Care.

[30] van Loon KA, Bonnie LHA, van Dijk N, Scheele F (2021). Benefits of EPAs at risk? The influence of the workplace environment on the uptake of EPAs in EPA-based curricula. Perspect Med Educ.

[31] van Loon KA, Scheele F (2021). Improving graduate medical education through faculty empowerment instead of detailed guidelines. Acad Med.

[32] Peters S, Bussières A, Depreitere B, Vanholle S, Cristens J, Vermandere M (2020). Facilitating guideline implementation in primary health care practices. J Prim Care Community Health.

[33] Peters S, Sukumar K, Blanchard S, Ramasamy A, Malinowski J, Ginex P (2022). Trends in guideline implementation: an updated scoping review. Implement Sci.

[34] Szulewski A, Braund H, Dagnone DJ, McEwen L, Dalgarno N, Schultz KW (2023). The assessment burden in competency-based medical education: how programs are adapting. Acad Med.

[35] Thaler RH (2018). Nudge, not sludge. Science.

[36] Schut S, Driessen E, van Tartwijk J, van der Vleuten C, Heeneman S (2018). Stakes in the eye of the beholder: an international study of learners’ perceptions within programmatic assessment. Med Educ.

[37] Watling CJ, Ginsburg S (2019). Assessment, feedback and the alchemy of learning. Med Educ.

[38] Gaunt A, Patel A, Rusius V, Royle TJ, Markham DH, Pawlikowska T (2017). 'Playing the game': how do surgical trainees seek feedback using workplace-based assessment?. Med Educ.

[39] Martin L, Sibbald M, Brandt Vegas D, Russell D, Govaerts M (2020). The impact of entrustment assessments on feedback and learning: trainee perspectives. Med Educ.

[40] Bray MJ, Bradley EB, Martindale JR, Gusic ME (2021). Implementing systematic faculty development to support an EPA-Based program of assessment: strategies, outcomes, and lessons learned. Teach Learn Med.

[41] Lövquist E, Shorten G, Aboulafia A (2012). Virtual reality-based medical training and assessment: the multidisciplinary relationship between clinicians, educators and developers. Med Teach.

[42] Norcini JJ (2003). Peer assessment of competence. Med Educ.

[43] Norcini J, Burch V (2007). Workplace-based assessment as an educational tool: AMEE guide no. 31. Med Teach.

[44] Slattery P, Saeri AK, Bragge P (2020). Research co-design in health: a rapid overview of reviews. Health Res Policy Syst.

